# Biochemical abnormalities among patients referred for celiac disease antibody blood testing in a primary health care setting

**DOI:** 10.1038/s41598-022-10492-6

**Published:** 2022-04-18

**Authors:** Line Lund Kårhus, Margit Kriegbaum, Mia Klinten Grand, Bent Struer Lind, Line Tang Møllehave, Jüri J. Rumessen, Christen Lykkegaard Andersen, Allan Linneberg

**Affiliations:** 1 Center for Clinical Research and Prevention, Copenhagen University Hospital – Bispebjerg and Frederiksberg, Frederiksberg, Copenhagen Denmark; 2 Copenhagen Primary Care Laboratory (CopLab) Database, Research Unit for General Practice and Section of General Practice, Department of Public Health, University of Copenhagen, Copenhagen, Denmark; 3 Department of Clinical Biochemistry, Copenhagen University Hospital Hvidovre, Hvidovre, Denmark; 4 Department of Gastroenterology, Copenhagen University Hospital - Herlev and Gentofte, Copenhagen, Denmark; 5 Department of Hematology, Rigshospitalet, Copenhagen, Denmark; 6 Department of Clinical Medicine, Faculty of Health and Medical Sciences, University of Copenhagen, Copenhagen, Denmark

**Keywords:** Coeliac disease, Biomarkers, Epidemiology

## Abstract

To investigate possible biochemical abnormalities associated with celiac disease (CD) antibody positivity in a primary health care setting and thereby identify predictors that could potentially reduce diagnostic delay and underdiagnosis of CD. This observational cohort study included measurements of CD antibodies in the Copenhagen Primary Care Laboratory (CopLab) database from 2000 to 2015; CD antibody positivity was defined as tissue transglutaminase antibody IgA or IgG ≥ 7 kU/L and/or deamidated gliadin peptide antibody IgG ≥ 10 kU/L. Individuals with a prior diagnosis of CD were excluded. We examined differences between individuals with positive and negative CD antibody tests regarding the results of biochemical tests performed six months before and one month after the date of the CD antibody test. We identified 76,265 measurements of CD antibodies during 2000–2015, and 57,061 individuals met the inclusion criteria (706 antibody-positive and 56,355 antibody-negative). We found lower ferritin, hemoglobin, cobalamin and folic acid levels and higher levels of transferrin, ALAT (alanine transaminase), and alkaline phosphate among individuals with a positive CD antibody test. Furthermore, we illustrated more measurements below the sex-specific reference intervals for hemoglobin, mean corpuscular volume (MCV), mean corpuscular hemoglobin concentration (MCHC), ferritin, cobalamin and folic acid among individuals with a positive CD antibody test. This study identified several biochemical abnormalities associated with CD antibody positivity among individuals referred to CD antibody testing. The pattern of abnormalities suggested that micronutrient deficiencies were prevalent among CD antibody-positive individuals, confirming malabsorption as a sign of CD. These findings illustrate the possibility of reducing diagnostic delay and underdiagnosis of CD.

## Introduction

Celiac disease (CD) is a chronic disease occurring in all age groups and affecting approximately 1% of the population^1^, although many cases of CD remain undiagnosed^2–8^. This condition is caused by an abnormal immune response in genetically susceptible individuals triggered by the ingestion of gluten proteins from wheat, rye and barley^2–4^.

Celiac disease primarily affects the small intestine, often leading to malabsorption and micronutrient deficiencies. A small intestinal biopsy with recognition of villus atrophy and inflammation has been the gold standard for diagnosis; however, serological testing is increasingly used in the diagnostic process and screening for CD, mainly by the detection of CD-specific antibodies, primarily immunoglobulin (Ig) A against tissue transglutaminase (TTG), the autoantigen in CD^5,9,10^. Management consists of a life-long gluten-free diet.

Screening for CD among individuals without typical symptoms—or even in the general population—remains controversial because many screen-detected cases have few or no symptoms, and little is known about the prognosis of undiagnosed CD. Nevertheless, studies suggest that asymptomatic patients with serological biomarkers indicative of CD may also benefit from a gluten-free diet^11,12^, and we have recently reported that undiagnosed CD may have an increased risk of adverse long-term consequences such as cancer and cardiovascular diseases^13^.

In Denmark, the prevalence of diagnosed CD is lower than in other European countries, despite recent increases in the prevalence of diagnosed cases as recorded in national registries^14,15^. Furthermore, we have previously shown that CD is markedly underdiagnosed in the general population: the prevalence found by screening (screening prevalence) was up to ten times the registered prevalence of diagnosed CD^16^. In line with other studies, we found no differences in symptoms between participants with and without screen-detected disease^17–19^. This suggests that screening for symptoms may not be an effective strategy for the detection of undiagnosed CD in a population setting. However, there is evidence that patients with CD often have hematological and other biochemical abnormalities at the time of diagnosis^20^. Furthermore, CD is known to be associated with malabsorption, especially before diagnosis, as evidenced by various deficiencies in nutrients, vitamins, minerals and trace elements, resulting in iron-deficiency anemia as well as calcium and vitamin B and D deficiencies^20–22^.

Diagnostic delay is a challenge in CD, and an average time to diagnosis of up to 10 years has been reported^23–26^. A Swedish study found that the mean delay to diagnosis was ten years from the first symptoms and six years from the first doctor’s visit^27^. The long diagnostic delay is a potential burden for both the patient and society, resulting in more health care contacts and costs, as well as costs for sick leave and decreased productivity. Casuistic reports indicate that the diagnostic delay is also significant in Denmark^28^, although we do not yet have survey data investigating the delay from first symptom/health care contact to a diagnosis of CD.

The overall aim of the present study was to describe the pattern of hematological and other biochemical test results among individuals referred for CD antibody blood testing in a primary health care setting in Denmark. In a primary health care setting, we aimed to investigate whether there were distinct hematological and/or other biochemical abnormalities associated with being CD antibody test positive and/or diagnosed with CD. Identification of hematological and/or biochemical abnormalities that predict CD will potentially enable us to reduce diagnostic delay and underdiagnosis of CD. This could, in turn, lead to early treatment and prevention of complications in patients with CD.

## Methods

### Data

#### The Copenhagen primary care laboratory (CopLab) database

This observational cohort study included results from primary care in the Copenhagen area of Denmark. In Denmark, citizens have direct access to primary care and hospital care at no cost. Approximately 98% of Danish citizens are listed with a general practitioner (GP), and Danish GPs are gatekeepers to more specialized patient care by specialized consultants as well as most in- and outpatient hospital care^29^. In the Copenhagen area (the Copenhagen Municipality and the former Copenhagen County), with its approximately 1.2 million inhabitants, only one laboratory served general practitioners and practicing specialists until 2015, the Copenhagen General Practitioners’ Laboratory (CGPL, *Københavns Praktiserende Lægers Laboratorium*). The CGPL provided a broad range of blood and urine biochemical tests, clinical physiological tests, and various cardiac tests and was accredited for International Organization for Standardization (ISO) standards ISO17025 and ISO15189. All data regarding the analyses performed since July 2000 have been saved in The Copenhagen Primary Care Laboratory (CopLab) database containing all results (n = 176,000,000) from July 1, 2000 to December 31, 2015 from the CGPL. Materials concerning the CopLab database have also been described elsewhere^30,31^.

For the present study, we used data from the CopLab database. The CopLab database contains information concerning the date of blood testing and thereby the age at antibody measurement. Celiac antibody tests performed prior to 2006 were externally analyzed, and since the database does not include numerical results from these tests, they were not included in the study population. However, information concerning the number of tests was used for Table 2, providing an overview of the distribution of tests in the study period.

#### National registers

In Denmark, all citizens are registered with a unique civil registration number, which enables person-level linkages across nationwide registers. The study population was linked to The Danish Civil Registration System^32^, which provides information on vital status; Statistics Denmark^33^, with information on income; Danish Education Registers^34^, which provides information on highest attained education; Danish National Health Register^35^, which provides information on the number of contacts with the health care system; and The Danish National Patient Register (NPR)^36^, which contains recorded information on all hospital contacts in Denmark since 1977, with individual diagnoses registered according to the World Health Organization’s (WHO) International Classification of Diseases (ICD- system)^37^.

For the present study, we used data from NPR from 1978 to 2018 and The Danish Civil Registration System until 2018. The ICD codes used for this study were the diagnosis codes for CD: ICD-8 269.00 and ICD-10: K90.0 to exclude individuals with a prior known diagnosis of CD from the study population. The age at antibody measurement and sex were derived from the unique civil registration number in the CopLab database. Education, income and country of origin were registered on January 1 in the year of CD measurement;

The *household equivalized income* takes into consideration the total income of the household as well as the composition of the household (number of adults and children). Hence, household equivalized income accurately reflects purchasing power. Information on equivalized household income was obtained from income registers at Statistics Denmark and was adjusted for inflation and related to the consumer price index in 2015. Income was recorded in Danish crowns (DKK) but displayed in Euros using the conversion rate from 2015 (100€ = 744 DKK).

*Educational attainment* was retrieved from the education registry at the year of the CD test and classified into three categories according to the International Standard Classification of Education (ISCED)-system (UNESCO 1997): up to 10 years of education = primary or lower secondary education (ISCED level 0–2), 11–12 years of education = upper secondary education (ISCED level 3) and 13 and more years of education = post-secondary and tertiary education (ISCED level 4–6). For individuals aged 24 or younger, information on the educational level of both parents was categorized as described above, and the individual was assigned the highest value of one’s own, the father’s and mother’s education.

*Country of origin* was derived from the population registry and grouped into Danish versus non-Danish (comprising migrants and descendants of migrants).

#### Biochemical blood tests

##### Celiac disease antibody tests

Tissue transglutaminase antibody (IgA) (TTG-IgA), tissue transglutaminase antibody (IgG) (TTG-IgG), deamidated gliadin peptide antibody (IgA) (DGP-IgA) and deamidated gliadin peptide antibody (IgG) (DGP-IgG) were measured in serum by fluorescence enzyme immunoassay (EIA) on the UniCAP 100 and ImmunoCAP 250 platforms (Phadia Laboratory Systems, Thermo Fisher Scientific, Hvidovre Denmark) according to the instructions of the manufacturer. For all four assays, the results were reported as negative (< 7 kU/L), equivocal (7–10 kU/L) or positive (> 10 kU/L). The results are valid from CGPL from July 13, 2006 (TTG-IgA) and July 1, 2013 (TTG-IgG, DGP-IgA and DGP-IgG). Before these dates, the CD antibodies were analyzed by the same method by an external collaborator, Phadia/Thermo Fisher, and the results were saved at CGPL. The interserial (day to day) coefficient of variation percentage determined on internal quality control material from the manufacturer was 17.3% (at level 0,28 kU/L, n = 1552) and 11.7% (at level 66,4 kU/L, n = 1545) for TTG-IgA; 23.6% (at level 0.117 kU/L n = 12) and 7.3% (at level 38.4 kU/L, n = 18) for TTG-IgG; 15.0% (at level 0.43 kU/L, n = 12) and 5.7% (at level 33.2 kU/L, n = 27) for DGP-IgA; and 34.6% (at level 0.042 kU/L, n = 12) and 8.6% (at level 65.2 kU/L, n = 28). The interserial (day to day) coefficient of variation determined on patient samples was estimated at 5–10% (at levels 7–10 kU/L), which indicates that the probability of a test result changing from negative to positive or vice versa due to random variation is highly unlikely (p < 0,01). The reportable ranges were 0.1–128 kU/L (TTG-IgA), 0.6–600 kU/L (TTG-IgG), 0.1–142 kU/L (DGP-IgA) and 0.4–302 kU/L (DGP-IgG). The four assays were subject to external quality control through participation in the UK NEQAS for COELIAC DISEASE external quality assessment service (Sheffield, United Kingdom). The assessment scheme included 6 distributions annually. Each distribution comprised 1 sample classified as either positive, negative or equivocal. The results from the UK NEQAS confirmed the reliability of the assay, and the samples were correctly classified in all cases for TTG-IgA (n = 54), TTG-IgG (n = 14) and DGP-IgA (n = 14). For DGP-IgG disease, 13 of 14 samples were correctly classified, and 1 equivocal sample was classified as positive.

In the present study, we defined, as in earlier studies^13,38^, CD antibody positivity as IgA TTG ≥ 7 kU/L, IgG TTG ≥ 7 kU/L and/or IgG DGP ≥ 10 kU/L.

##### Other hematological and biochemical tests

Other variables used from the CopLab database were hemoglobin, MCV (erythrocytes, mean corpuscular volume), MCHC (mean corpuscular hemoglobin concentration), transferrin, hematocrit, ferritin, ALAT (alanine transaminase), alkaline phosphate, 25-OH vitamin D, folic acid, cobalamin, CRP (C-reactive protein), ReticMCH (reticulocyte, mean corpuscular hemoglobin), RDW (erythrocyte volume, relative distribution width), and immunoglobulin A. A basic characterization of other blood tests is summarized in Additional Table S1 (Additional file 1). Reference intervals used to define abnormal values are presented in Additional Table S2 (Additional file 1).

### Study population

**Figure 1:**
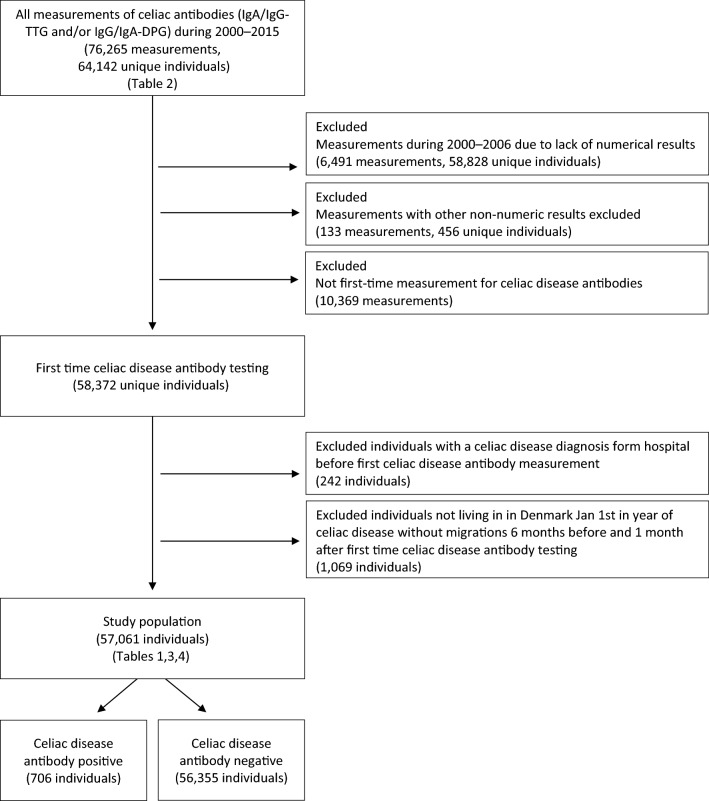
Flow diagram, description of the selection of the study population.

The flow chart in Fig. 1 depicts the study population, which comprised all individuals referred to CGPL during 2000–2015, both from GPs and practicing specialists, for measurement of CD antibodies: IgA- and IgG-TTG and IgG and IgG deamidated gliadin (DGP). The CopLab database included a total of 76,265 blood sample analyses with at least one celiac antibody analysis during 2000–2015. As mentioned under “Data”, CD antibody tests performed prior to 2006 were externally analyzed, and since the database does not include numerical results from these tests, they were not included in the study population. However, the numbers of tests were used for Table 2, giving an overview of the distribution of tests in the study period. The study population ultimately consisted of individuals with a first-time CD antibody measurement who were living in Denmark on January 1 of the year of CD measurement without migrations six months before and one month after the first CD antibody testing. Individuals with a prior diagnosis ICD-8/10 code for CD in the registries were excluded.

### Statistics

#### Differences in baseline characteristics between CD antibody-positive and CD antibody-negative patients and trends in requisitions (Tables 1 and 2)

The distribution of categorical covariates was summarized using counts and percentages. Continuous covariates with a nonnormal distribution were summarized using the median and interquartile range (IQR).

In Table 1, chi-square tests were used to test differences between categorical variables. For continuous variables (age and income), the Wilcoxon test was used, as none of the included variables were normally distributed. Two-sided p values were reported for all tests. The *p* values were adjusted for multiple testing, and values < 0.05 were considered statistically significant^39^.

**Table 1:** Characteristics of individuals with positive and negative celiac disease antibody test results.

	Celiac disease antibody positive^a^	Celiac disease antibody negative^a^	*P*-value^b^
n	706	56,355	
Sex (percent female)	72.8%	66.2%	< 0.001
Age median (IQR)	26 (13–40)	29 (20–44)	< 0.001
Country of origin^c^			0.056
Danish	599 (84.8%)	46,155 (81.9%)	
Not Danish	107 (15.2%)	10,200 (18.1%)	
**Education**			
Unknown	22 (3.1%)	1,613 (2.9%)	0.003
Low	51 (7.2%)	6,253 (11.1%)	
Medium	211 (29.9%)	18,425 (32.7%)	
High	422 (59.8%)	30,064 (53.3%)	
Income median (IQR)^d^	32,052.9 (21,858.0–43,031.8)	29,770.8 (20,229.5–41,433.8)	0.003
Number of contacts with primary health care five years before celiac disease measurement, median (IQR)^e^	62 (36–93)	65 (38–105)	0.056

*IQR* Interquartile range.

^a^positive celiac antibody test was defined as: IgA/IgG TTG of at least 7 U/ml or IgG DGP of at least 10 U/ml and a negative test was defined as IgA /IgG TTG under 7 U/ml and IgG DGP under 10 U/ml.

^b^Chi square tests were used to test differences between categorical variables. For continuous, (age and income) Wilcoxon test was used (age and income were not normally distributed). 2-sided p-values were reported for all tests. P-values were adjusted for multiple testing^39^.

^c^Origin was derived from the population registry and grouped into Danish vs. non-Danish (comprising migrants and descendants of migrants).

^d^The household equivalized income in Euros. Income was missing for 361 individuals.

^e^The total number of contacts with primary health care, recorded from the reimbursement system the five years before celiac disease antibody measurement.

#### Associations with biochemical biomarkers (Tables 3 and 4)

The distribution of categorical covariates was estimated using counts and percentages. Continuous covariates with a log-normal distribution were summarized using the median, calculated as the exponential of the mean of the log-transformed values which is also known as the geometric mean^40^. The 95% confidence intervals were calculated using a robust variance estimator to account for dependence between tests from the same person^41^. Only for CRP was a parametric survival model with Gaussian errors and a robust variance estimator used to account for the left censoring due to lower limits of detection^42^.

### Ethics approval and consent to participate

Ethical and data handling approval were obtained by the Faculty of Health Science, University of Copenhagen (case no. 514-0460/20-3000). According to Danish legislation, no ethical approval or consent is required for registry-based research projects, in which individuals included in the study are not approached at any time during the conduct of the study.

## Results

The selection of individuals in the study is shown in Fig. 1, resulting in a population of 706 CD antibody-positive and 56,355 CD antibody-negative individuals.

We compared the characteristics of CD antibody-positive individuals with those of CD antibody-negative individuals (Table 1). We found that the antibody-positive individuals were younger, and a larger percentage were women. There was no statistically significant difference in the number of contacts with primary health care during the five-year period prior to CD antibody measurement, but we observed differences with regard to country of origin, education, and income (Table 1); however, these differences were small. Trends in the frequency of requisitions for CD antibody tests from primary care per calendar year are illustrated in Table 2. There was a clear and continuously increasing trend in the number of persons tested throughout the observed 15-year period, and more women than men were tested. The median age of persons tested was approximately 30 throughout the study period, varying from a median age of 26 to a median age of 32 when calculated per year.

**Table 2:** Trends in frequency of requisitions of celiac disease antibody tests from primary care, both general practitioners (GP) and private practicing specialists, from 2000 to 2015*.*

Year	Number of tests	IgA-TTG		IgG-TTG		IgA-DGP		IgG-DGP		Number of persons		Age median (IQR)	Percent female
		Total primary care	GP	Total primary care	GP	Total primary care	GP	Total primary care	GP	Total primary care	GP		
2000	156	156	25	.	.	.	.	.	.	156	25	26 (6.50;41.0)	61.5
2001	334	334	96	.	.	.	.	.	.	329	95	27 (9.00;44.0)	61.7
2002	384	384	133	.	.	.	.	.	.	378	132	31 (14.0;46.0)	63.2
2003	1204	1186	488	18	< 5	.	.	.	.	1171	486	30 (19.0;45.0)	65.8
2004	1466	1435	540	31	< 5	.	.	.	.	1427	534	30 (20.0;45.0)	66.0
2005	1883	1862	749	21	< 5	.	.	.	.	1836	742	32 (22.0;47.0)	64.6
2006	2139	2088	913	51	9	.	.	.	.	2035	885	30 (21.0;45.0)	66.9
2007	2567	2524	1089	43	15	.	.	.	.	2384	1001	30 (20.0;43.0)	65.6
2008	3953	3877	1784	76	25	.	.	.	.	3744	1740	31 (20.0;46.0)	66.7
2009	4655	4583	2074	72	26	.	.	.	.	4457	2030	30 (20.0;44.0)	67.1
2010	5234	5171	2322	63	24	.	.	.	.	5029	2273	30 (20.0;45.0)	65.4
2011	5938	5864	2841	74	36	.	.	.	.	5676	2778	29 (19.0;44.0)	65.6
2012	7698	7587	4187	111	59	.	.	.	.	7338	4111	29 (20.0;43.0)	66.2
2013	10,515	9848	5687	133	85	268	108	266	105	9512	5582	29 (20.0;44.0)	67.0
2014	13,440	12,101	7610	214	137	561	255	564	258	11,689	7437	29 (20.0;44.0)	66.7
2015	14,699	13,072	8442	212	136	689	311	726	317	12,663	8309	29 (19.0;43.0)	66.0

*Ig* Immunoglobulin, *TTG* Tissue transglutaminase, *DGP* Deamidated gliadin peptide, *GP* General practitioner, *IQR* Interquartile range.

Table 3 depicts differences in median levels of hematological and other biochemical biomarkers between individuals, referred to the laboratory, with positive and negative CD antibody tests stratified by sex. The most remarkable difference was, for both men and women, the markedly lower ferritin among the CD antibody-positive individuals compared with the CD antibody-negative individuals; for women 13.8 versus 35.9 µg/L and for men 34.3 versus 80.4 µg/L, respectively. We also found a tendency of lower hemoglobin among CD antibody-positive individuals; for women, 7.8 versus 8.1 mmol/L; and for men, 8.5 versus 8.8 mmol/L, respectively. Furthermore, we observed lower cobalamin and folic acid levels, while higher levels of transferrin, ALAT and alkaline phosphate were noted among CD antibody-positive men and women.

**Table 3:** Results of hematological and biochemical measurements during the period six months before to one month after blood drawing and testing among individuals with positive and negative celiac antibody tests.

	Number of tests	Percentage with measurement (%)	*Women*		*Men*	
			Celiac antibody positive^a^ women N = 4,613 tests in 513 women Median (95% CI)	Celiac antibody negative^a^ women N = 309,658 tests in 37,218 women Median (95% CI)	Celiac antibody positive^a^ men N = 1,497 tests in 192 men Median (95% CI)	Celiac antibody negative^a^ men N = 153,827 tests in 19,019 men Median (95% CI)
Hemoglobin (mmol/L)	58,883	81.7	7.8 (7.8–7.9)	8.1 (8.1–8.1)	8.5 (8.4–8.7)	8.8 (8.8–8.8)
MCV ((Erythrocytes, mean corpuscular volume) (fL)	58,883	81.7	86.6 (85.9–87.4)	88.6 (88.6–88.7)	85.8 (84.7–87.0)	86.8 (86.7–86.9)
MCHC (Mean corpuscular hemoglobin concentration) (mmol/L)	14,044	22.4	20.3 (20.1–20.4)	20.7 (20.7–20.7)	20.8 (20.6–21.1)	21.0 (21.0–21.0)
Transferrin (µmol/L)	8,000	13.3	37.5 (35.7–39.3)	34.2 (34.0–34.3)	32.9 (31.2–34.7)	31.4 (31.2–31.6)
Transferrin (Fe-binding sites; P)—Iron	7,527	12.6	0.1 (0.1–0.1)	0.2 (0.2–0.2)	0.2 (0.2–0.3)	0.2 (0.2–0.2)
Ferritin (µg/L)	13,549	21.5	13.7 (11.3–16.7)	35.9 (35.2–36.6)	34.3 (25.2–46.8)	80.4 (78.0–83.0)
ALAT (Alanine transaminase) (U/L)	47,751	68.8	22.6 (21.6–23.6)	19.8 (19.7–19.9)	28.0 (25.9–30.3)	25.3 (25,1–25,5)
Alkaline phosphate (U/L)	43,964	63.9	87.2 (81.7–93.2)	74.1 (73.6–74.6)	113.3 (101.8–126.1)	100.1 (99.0–101.2)
Vitamin D (nmol/L)	28,599	43.8	60.3 (56.8–64.1)	58.9 (58.5–59.3)	66.8 (61.4–72.6)	55.1 (54.5–55.7)
ReticMCH (Reticulocyte, mean corpuscular hemoglobin) (fmol)	2,472	3.9	1.7 (1.6–1.7)	1.8 (1.8–1.9)	1.8 (1.8–1.9)	1.8 (1.8–1.9)
RDW (Erythrocytes volume, relative distribution width) (%)	58,882	81.7	13.8 (13.6–13.9)	13.2 (13.1–13.2)	13.5 (13.3–13.6)	13.2 (13.2–13.2)
Immunoglobulin A (g/L)	58,125	98.7	1.9 (1.8–2.0)	1.8 (1.8–1.8)	1.6 (1.5–1.8)	1.8 (1.8–1.8)
Cobalamin (pmol/L)	19,886	31.8	280.1 (261.2–300.4)	289.1 (287.1–291.2)	270.0 (244.5–298.1)	306.0 (303.1–308.8)
Folic acid (nmol/L)	2,839	4.7	12.9 (10.5–16.0)	17.5 (17.0–18.0)	8.9 (4.0–19.7)	16.5 (15.9–17.1)
C-reactive protein (CRP) (mg/L) ^b^ including <	46,205	66.6	0.9 (0.6–1.3)	1.2 (1.1–1.2)	0.7 (0.3–1.4)	0.6 (0.5–0.6)

^a^A positive celiac antibody test was defined as: IgA/IgG TTG of at least 7 U/ml or IgG DGP of at least 10 U/ml and a negative test was defined as IgA /IgG TTG under 7 U/ml and IgG DGP under 10 U/ml.

^b^CRP was reported with ‘ < ’ in many cases; Low results were reported as < 5 mg/L (between December 2, 2002 and May 28, 2008) and < 4 (from May 29, 2008).

Table 4 presents, for each hematological and other biochemical biomarker, the proportion of tests with biomarker measurements outside (below or above) sex-specific reference intervals for each measurement among individuals, referred to CD antibody testing, with positive and negative CD antibodies. We found that a greater proportion of tests among the CD antibody-positive individuals compared with negative individuals exhibited hemoglobin (10.2% vs. 2.7%), MCV (7.1% vs. 2.9%), MCHC (6.8% vs. 1.2%), and ferritin (37.6% vs. 7.6%) below, while transferrin (20.7% vs. 9.5%) was above the reference interval. Furthermore, deficiency of cobalamin and folic acid was more common among tests from individuals with positive CD antibodies.

**Table 4:** Results of hematological and biochemical biomarker measurements during the period six months prior to one month after celiac disease antibody measurement among individuals with positive and negative celiac antibody tests^a^. Test results are defined as below or above sex-specific reference intervals for each measurement of the biomarker^b^.

	Celiac antibody positive ^a^ individuals			Celiac antibody negative^a^ individuals		
	Number of tests	Tests below reference interval % (95% CI)	Tests above reference interval % (95% CI)	Number of tests	Tests below reference interval % (95% CI)	Tests above reference interval % (95% CI)
Hemoglobin	752	10.2 (8–12.8)	< 5^c^	58,131	2.7 (2.6–2.9)	0.3 (0.3–0.4)
MCV (Erythrocytes. mean corpuscular volume)	752	7.1 (5.3–9.4)	2.2 (1.3–3.7)	58,131	2.9 (2.8–3.1)	1.3 (1.0–1.4)
MCHC (Mean corpuscular hemoglobin concentration)	196	6.8 (4.0–11.3)	< 5^c^	13,848	1.2 (1.0–1.4)	4.8 (4.4–5.1)
Transferrin	111	< 5^c^	20.7 (14.1–29.3)	7,889	1.5 (1.3–1.8)	9.5 (8.8–10.2)
Transferrin (Fe-binding sites; P)—Iron	107	33.8 (25.2–43.7)	< 5^c^	7,420	13.8 (13.1–14.6)	2.2 (1.9–2.6)
Ferritin	239	37.6 (31.2–44.5)	< 5^c^	13,310	7.6 (7.1–8.0)	4.1 (3.8–4.5)
ALAT (Alanine transaminase)	590	< 5^c^	1.2 (0.6–2.5)	47,161	0.5 (0.5–0.6)	1.5 (1.4–1.6)
Alkaline phosphate	556	1.9 (1.0–3.6)	5.3 (3.6–7.7)	43,408	2 (1.9–2.2)	4.2 (4.0–4.4)
Vitamin D	379	28.3 (23.9–33.2)	< 5^c^	28,206	31.7 (31.1–32.3)	0.1 (0.1–0.2)
ReticMCH (Reticulocyte, mean corpuscular hemoglobin)	56	45.1 (32.1–58.7)	< 5^c^	2,416	19.9 (18.3–21.6))	2.1 (1.6–2.8)
RDW (Erythrocytes volume, relative distribution width)	752	< 5^c^	14.4 (11.8–17.5)	58,130	< 5 ^c^	3.7 (3.5–3.9)
Immunoglobulin A	727	1.4 (0.7–2.5)	3.7 (2.6–5.4)	57,398	1.2 (1.1–1.3)	2.4 (2.2–2.5)

	Number of tests	Tests below signal value^d^ % (95% CI)	Tests above signal value^d^ % (95% CI)	Number of tests	Tests below signal value^d^ % (95% CI)	Tests above signal value^d^ % (95% CI)
Cobalamin	269	9.0 (6.1–13.1)^d^	Na^d^	19,617	3.5 (3.2–3.7)^d^	na^d^
Folic acid	67	20.4 (12.2–32.1)^d^	na^d^	2,772	4.3 (3.6–5.1)^d^	na^d^
C-reactive protein (CRP)	557	na^d^	5.4 (3.8–7.7)^d^	45,648	na^d^	7.0 (6.8–7.3)^d^

^b^See Additional Table S2 (Additional file 1) for reference intervals or signal values.

^a^A positive celiac antibody test was defined as: IgA/IgG TTG of at least 7 U/ml or IgG DGP of at least 10 U/ml and a negative test was defined as IgA /IgG TTG under 7 U/ml and IgG DGP under 10 U/ml.

^c^Results where the total number of observations was under 5 are not shown due to anonymization.

^d^For Cobalamin, Folic acid and CRP the results are listed with signal values and not reference interval. Therefore, results below the signal value for folic acid and cobalamin are considered as abnormal and for CRP results above the signal value are considered as abnormal.

*CI* Confidence interval; *NA* not applicable.

## Discussion

This study has revealed relevant prediagnostic differences in hematological and other biochemical test results between individuals with positive and negative CD antibodies among individuals referred to the laboratory for celiac disease antibody measurements. Thus, individuals with positive CD antibodies were more likely to have blood levels of hemoglobin, MCV, MCHC, ferritin, cobalamin and folic acid below and transferrin above the reference interval. These results suggest that hematological and other biochemical abnormalities may be important markers of undiagnosed CD.

Overall, the pattern of these biomarkers suggests malabsorption, for example iron deficiency anemia, among individuals with positive CD antibodies. CD is known to be associated with malabsorption, especially before diagnosis, as exemplified by various deficiencies of micronutrients, resulting in iron-deficiency anemia and vitamin B and D deficiencies, among others^21,22^. Even though malabsorption is described as a classic sign of CD^6,23^, studies have found malabsorption to be associated with an increased risk of a long delay of diagnosis^24,26^. This study confirms the association of CD and malabsorption, as we observed more measurements below the reference interval for hemoglobin, ferritin, cobalamin and folic acid among individuals referred to CD antibody testing with positive CD antibodies. These results confirm the potential of screening for CD among individuals with specific laboratory signs of malabsorption. However, the results are not adjusted for age and we do not have information on mineral or vitamin supplements, which might affect the results, and the association could likely be stronger if this adjustment had been possible. Furthermore, several measurements could be included form the same individual, e.g. controls for abnormal results, but we used calculated values taking repeated measurements into account in the statistical methods.

The increase in CD antibody measurements during 2000–2015 corresponds to the increase in CD diagnoses seen in the Danish registers^14,15^, as well as to the increase observed internationally^20^. From 2013, the DGP tests were introduced, but not all individuals were tested with both TTG and DGP. There were some combined tests that could be requested; from 2003, a ‘CD test’ included both TTG-IgA and total IgA, and an algorithm resulted in a test for TTG-IgG if both TTG-IgA and IgA were low. Starting in 2013, if TTG-IgG was low in children, both DGP-IgA and DGP-IgG were measured. These algorithms might have influenced the measurements, possibly resulting in more awareness among the GPs and an increase in the number of tests per person, but the physicians could also request separate CD antibodies. The number and type of CD antibody measurements are consistent with the acceptance and considerable evidence supporting the use of TTG-IgA assays as the first-line test for the diagnosis of CD^20^. More women than men were tested for CD, and these numbers are in line with the notion that more women than men are diagnosed with CD^15^ but also in line with the knowledge of an increased risk for CD among girls and women^43^.

A strength of this study is that it includes data from primary care, which is the most important setting for identifying persons in need of diagnostic work-up. This is a unique possibility to describe patterns of CD antibody measurements requested from primary health care. It is also a strength that the same methods for CD antibody measurement were used throughout the study period and that we had the results of hematological and other biochemical tests even before the CD measurements were performed at CGPL. The possibility of linkage to the Danish national registers is a strength for comparing the groups. Moreover, it is a strength that the CopLab database includes all blood samples from the Copenhagen area for primary care, resulting in a large number of tests and individuals. Nevertheless, as the prevalence of CD is so low, even with this large number of individuals, the number of CD antibody-positive individuals was small. It is also important to note, that the results in Table 4 are presented by number of tests, and the same individual potentially could have several measurements included, e.g. controls for abnormal results. However, the percentages and 95% confidence intervals are calculated taking repeated measurements into account. Furthermore, the population is highly selected, as they are referred to the laboratory for a CD antibody test, and individuals with other biomarker measurements might be a further selected group. Therefore, the results of this highly selected population of seropositive individuals cannot be generalized to all seropositive cases. Moreover, the control group should not be considered healthy controls, as there might be other reasons for their tests, and we do not know the reason for referral to the laboratory. For example, deficiencies could be a symptom/condition causing testing for CD antibodies and may thereby affect the number of tests, especially iron-deficiency anemia, as guidelines recommend screening for CD if iron-deficiency anemia is unexplained^44,45^. It is important to note that diagnoses in primary care, e.g., privately practicing gastroenterologists, are not listed in the NPR; therefore, not all diagnosed CD cases are known, and some antibody-negative individuals could have a diagnosis of CD.

## Conclusion

This study identified several biochemical abnormalities associated with CD antibody positivity in a primary care setting, among individuals referred to CD antibody testing. The present study shows more measurements below the reference interval for hemoglobin, MCV, MCHC, ferritin, cobalamin and folic acid among the individuals with a positive CD antibody test. The pattern of the included biomarkers suggested that micronutrient deficiencies were common among CD antibody-positive individuals and confirmed malabsorption as a sign of CD. These findings illustrate the possibility of prospective development of biochemical algorithms to improve guidelines for CD screening to reduce diagnostic delay and underdiagnosis of CD and to lead to early treatment and prevention of comorbidities in patients with CD.

## Supplementary Information

Supplementary Information.

## Data Availability

The dataset supporting the conclusions of this article are based on data from Danish national health registers and restrictions apply to the availability of these data, which were used under license for the current study, and according to Danish law, this information cannot be publicly available. A request for access to the data needs approval from appropriate Danish authorities and are subject to Danish regulations on personal data protection.

## Abbreviations

ALAT: Alanine transaminase
CD: Celiac disease
CI: Confidence interval
CGPL: Copenhagen General Practitioners’ Laboratory
CopLab: Copenhagen Primary Care Laboratory
CRP: C-reactive protein
DKK: Danish crowns
DGP: Deamidated gliadin peptide
RDW: Erythrocyte volume, relative distribution width
GP: General practitioner
Ig: Immunoglobulin
ICD: International classification of disease
ISCED: International Standard Classification of Education
IQR: Interquartile range
ISO: International Organization for Standardization
MCHC: Mean corpuscular hemoglobin concentration
MCV: Mean corpuscular volume
ReticMCH: Reticulocyte, mean corpuscular hemoglobin
NPR: The Danish National Patient Register
TTG: Tissue transglutaminase
WHO: World Health Organization
