# Supplementary material for: Biochemical abnormalities among patients referred for celiac disease antibody blood testing in a primary health care setting
Source: Sci Rep. 2022 Apr 18;12:6407. doi: 10.1038/s41598-022-10492-6 (PMC9016061; doi:10.1038/s41598-022-10492-6)
Supplement: Supplementary file 1 — Supplementary Information. [file 41598_2022_10492_MOESM1_ESM.docx]

**ADDITIONAL FILE 1**

**Biochemical abnormalities among patients referred for celiac disease antibody blood testing in a primary health care setting**

Line Lund Kårhus, Margit Kriegbaum, Mia Klinten Grand, Bent Struer Lind, Line Tang Møllehave, Jüri J. Rumessen, Christen Lykkegaard Andersen, Allan Linneberg

**Contents**

Additional Table S1: *Characterization of supplementary blood tests*

Additional Table S2: *Reference values (or signal values) used for the supplementary blood tests*

| **Additional Table S1 Characterization of supplementary blood tests** | | | |
| --- | --- | --- | --- |
| Test | IUPAC code | Unit | Note |
| Hemoglobin | NPU02319 | mmol/L | 1 |
| Erythrocytes, mean corpuscular volume | NPU01944 | fL | 1 |
| Hemoglobin, MCHC | NPU02321 | mmol/L | 1 |
| Iron | NPU02508 | µmol/L | 2 |
| Transferrin | NPU03607 | µmol/L | 2 |
| Transferrin(Fe-binding sites; P)—Iron | NPU04191 | 1 | 3 |
| Erythrocytes; vol.fr. | NPU01961 | 1 | 1 |
| Ferritin | NPU19763 | µg/L | 2 |
| Alanine transaminase | NPU19651 | U/L | 2 |
| Alkaline phosphatase | NPU19655 | U/L | 2 |
| Vitamin D | NPU10267 | nmol/L | 2 |
| Rtcs(B)—Hemoglobin(Fe) | NPU17007 | fmol | 1 |
| Erythrocytes volume, relative distribution width | NPU18162 | % | 1 |
| Immunoglobulin A | NPU19795 | g/L | 2 |
| Cobalamin | NPU01700 | pmol/L | 4 |
| Folic acid | NPU02070 | nmol/L | 4 |
| C-reactive protein | NPU19748 | mg/L | 2 |
| Abbreviations: IUPAC, International Union of Pure and Applied Chemistry; NPU, Nomenclature for Properties and Units | | | |
| 1) Measured as described (Clin Epidemiol. 2014;6:199-211) | |  |  |
| 2) Measured as described (BMJ Open. 2020;10(5):e034318) | |  |  |
| 3) Calculated as [Iron]/(2*[Transferrin]) |  |  |  |
| 4) Measured in serum by the commercially available chemiluminescence immunoassays on the Centaur/Centaur XP platform (Bayer, Siemens, Healthcare Diagnostics, Tarrytown, NY, USA) | | | |

| **Additional Table S2 Reference values (or signal values) used for the supplementary blood tests** | | | | | | |  |
| --- | --- | --- | --- | --- | --- | --- | --- |
|  | Patient category | | |  | Reference values ^a^ | | |
| Test | Sex | Age unit | Age range |  | Lower limit | Upper limit | |
| Hemoglobin | Male | Year | 14 – 125 |  | 8 | 11 | |
| Hemoglobin | Female | Year | 14 – 125 |  | 7 | 10 | |
| Hemoglobin | All | Year | 8 –14 |  | 6.9 | 9.8 | |
| Hemoglobin | All | Year | 5 – 8 |  | 6.7 | 9.1 | |
| Hemoglobin | All | Year | 0 – 5 |  | 6.5 | 8.4 | |
| Hemoglobin | All | Day | 58 – 365 |  | 6.2 | 9 | |
| Hemoglobin | All | Day | 30 – 57 |  | 5.6 | 10.7 | |
| Hemoglobin | All | Day | 16 – 29 |  | 6.7 | 11.5 | |
| Hemoglobin | All | Day | 0 – 15 |  | 8.4 | 15.3 | |
|  |  |  |  |  |  |  | |
| Erythrocytes, mean corpuscular volume | All | Year | 14 – 125 |  | 80 | 100 | |
| Erythrocytes, mean corpuscular volume | All | Year | 8 – 14 |  | 76 | 97 | |
| Erythrocytes, mean corpuscular volume | All | Year | 0 – 8 |  | 72 | 90 | |
| Erythrocytes, mean corpuscular volume | All | Day | 58 – 365 |  | 74 | 98 | |
| Erythrocytes, mean corpuscular volume | All | Day | 16 – 57 |  | 82 | 110 | |
| Erythrocytes, mean corpuscular volume | All | Day | 0 – 15 |  | 94 | 118 | |
|  |  |  |  |  |  |  | |
| Hemoglobin, MCHC | All | Year | 5 – 125 |  | 19.0 | 22.0 | |
| Hemoglobin, MCHC | All | Year | 0 – 5 |  | 16.8 | 20.5 | |
| Hemoglobin, MCHC | All | Day | 0 – 56 |  | 16.0 | 22.0 | |
|  |  |  |  |  |  |  | |
| Iron | All | Year | 13 – 125 |  | 9 | 34 | |
| Iron | All | Year | 0 – 13 |  | 5 | 28 | |
| Iron | All | Day | 16 – 60 |  | 10 | 36 | |
| Iron | All | Day | 0 – 15 |  | 27 | 36 | |
|  |  |  |  |  |  |  | |
| Transferrin | All | Year | 14 – 125 |  | 24 | 41 | |
| Transferrin | All | Year | 0 – 14 |  | 19 | 50 | |
| Transferrin | All | Month | 6 – 12 |  | 18 | 46 | |
| Transferrin | All | Month | 0 – 6 |  | 13 | 41 | |
| Transferrin | All | Day | 0 – 30 |  | 12 | 26 | |
|  |  |  |  |  |  |  | |
| Transferrin(Fe bindingsites;P-Iron | Male | Year | 15 – 125 |  | 0.16 | 0.57 | |
| Transferrin(Fe bindingsites;P-Iron | Female | Year | 50 – 125 |  | 0.14 | 0.50 | |
| Transferrin(Fe bindingsites;P-Iron | Female | Year | 15 – 125 |  | 0.11 | 0.50 | |
| Transferrin(Fe bindingsites;P-Iron | All |  | 0 – 15 |  | 0.11 | 0.40 | |
|  |  |  |  |  |  |  | |
| Erythrocytes; vol.fr. | Male | Year | 14 – 125 |  | 0.40 | 0.52 | |
| Erythrocytes; vol.fr. | Male | Year | 7 – 14 |  | 0.35 | 0.47 | |
| Erythrocytes; vol.fr. | Female | Year | 7 – 125 |  | 0.35 | 0.47 | |
| Erythrocytes; vol.fr. | All | Year | 0 – 7 |  | 0.35 | 0.44 | |
| Erythrocytes; vol.fr. | All | Day | 58 – 180 |  | 0.32 | 0.46 | |
| Erythrocytes; vol.fr. | All | Day | 30 – 57 |  | 0.30 | 0.54 | |
| Erythrocytes; vol.fr. | All | Day | 16 – 29 |  | 0.42 | 0.62 | |
| Erythrocytes; vol.fr. | All | Day | 0 – 15 |  | 0.50 | 0.82 | |
|  |  |  |  |  |  |  | |
| Ferritin | All | Year | 6 – 125 |  | 12 | 300 | |
| Ferritin | All | Year | 0 – 6 |  | 6 | 60 | |
| Ferritin | All | Day | 182 – 360 |  | 6 | 45 | |
| Ferritin | All | Day | 32 – 181 |  | 6 | 340 | |
| Ferritin | All | Day | 0 - 31 |  | 6 | 515 | |
|  |  |  |  |  |  |  | |
| Alanine transaminase | Male | Year | 18 – 125 |  | 10 | 70 | |
| Alanine transaminase | Female | Year | 18 – 125 |  | 10 | 45 | |
| Alanine transaminase | All | Year | 0 – 18 |  | 10 | 45 | |
| Alkaline phosphatase | All | Year | 18 – 125 |  | 35 | 105 | |
| Alkaline phosphatase | Male | Year | 16 – 18 |  | 60 | 235 | |
| Alkaline phosphatase | Male | Year | 14 – 16 |  | 115 | 485 | |
| Alkaline phosphatase | Male | Year | 12 – 14 |  | 180 | 455 | |
| Alkaline phosphatase | Female | Year | 16 – 18 |  | 45 | 115 | |
| Alkaline phosphatase | Female | Year | 14 – 16 |  | 60 | 210 | |
| Alkaline phosphatase | Female | Year | 12 – 14 |  | 90 | 385 | |
| Alkaline phosphatase | All | Year | 10 – 12 |  | 115 | 515 | |
| Alkaline phosphatase | All | Year | 1 – 10 |  | 130 | 385 | |
| Alkaline phosphatase | All | Year | 0 – 1 |  | 55 | 425 | |
| Alkaline phosphatase | All | Day | 0 – 8 |  | 65 | 270 | |
|  |  |  |  |  |  |  | |
| Vitamin D ^b^ | All | Year | 0 – 125 |  | 50 | 200 | |
|  |  |  |  |  |  |  | |
| Rtcs(B)—Haemoglobin(Fe) | All | Year | 0 – 125 |  | 1.72 | 2.17 | |
|  |  |  |  |  |  |  | |
| Erythrocytes volume, relative distribution width | All | Year | 0 – 125 |  | 11 | 15 | |
|  |  |  |  |  |  |  | |
| Immunoglobulin A | All | Year | 18 – 125 |  | 0.70 | 4.30 | |
| Immunoglobulin A | Female | Year | 14 – 18 |  | 0.65 | 3.06 | |
| Immunoglobulin A | Female | Year | 10 – 14 |  | 0.53 | 2.93 | |
| Immunoglobulin A | Female | Year | 6 – 10 |  | 0.38 | 2.68 | |
| Immunoglobulin A | Female | Year | 4 – 6 |  | 0.30 | 2.22 | |
| Immunoglobulin A | Female | Year | 2 – 4 |  | 0.21 | 1.88 | |
| Immunoglobulin A | Female | Year | 0 – 2 |  | 0.14 | 1.44 | |
| Immunoglobulin A | Male | Year | 14 – 18 |  | 0.63 | 3.21 | |
| Immunoglobulin A | Male | Year | 10 – 14 |  | 0.52 | 3.04 | |
| Immunoglobulin A | Male | Year | 6 – 10 |  | 0.39 | 2.74 | |
| Immunoglobulin A | Male | Year | 4 – 6 |  | 0.30 | 2.22 | |
| Immunoglobulin A | Male | Year | 2 – 4 |  | 0.21 | 1.85 | |
| Immunoglobulin A | Male | Year | 0 – 2 |  | 0.14 | 1.37 | |
| Immunoglobulin A | All | Month | 6 – 12 |  | 0.08 | 0.91 | |
| Immunoglobulin A | All | Month | 0 – 6 |  | 0.05 | 0.57 | |
| Immunoglobulin A | All | Day | 0 – 90 |  | 0.01 | 0.34 | |
|  |  |  |  |  |  |  | |
|  | Patient category | | |  | Signal values ^c^ | | |
| Test | Sex | Age unit | Age range |  |  |  |  |
| Cobalamin | All | Year | 0 – 125 |  | ≥ 150 | | |
|  |  |  |  |  |  |  | |
| Folic acid | All | Year | 0 – 125 |  | ≥ 6 | | |
|  |  |  |  |  |  |  | |
| C-reactive protein | All | Year | 0 – 125 |  | ≤ 10 | | |
| ^a^ Except for Vitamin D, Cobalamin, Folic acid and C-reactive protein all reference values are health related values e.g. it is expected to include 95 % of results from healthy individuals. For units of the specific tests see Additional Table S1.  ^b^ The limits for Vitamin D are decision limits.  ^c^ For Cobalamin, Folic acid and C-reactive protein (CRP) the limits listed are signal values, hence for Cobalamin and Folic acid results under the signal value are considered as abnormal and for CRP results over the signal value 10 are considered as abnormal. | | | | | | |  |
